# A Better Cardiopulmonary Fitness Is Associated with Improved Concentration Level and Health-Related Quality of Life in Primary School Children

**DOI:** 10.3390/jcm11051326

**Published:** 2022-02-28

**Authors:** Katharina Köble, Tanja Postler, Renate Oberhoffer-Fritz, Thorsten Schulz

**Affiliations:** Institute of Preventive Pediatrics, Technical University of Munich, 80992 Munich, Germany; tanja.postler@tum.de (T.P.); renate.oberhoffer@tum.de (R.O.-F.); thorsten.schulz@tum.de (T.S.)

**Keywords:** cardiovascular health, fitness, elementary school, selective attention, psychosocial health

## Abstract

This study aimed to examine the physical fitness (PF) levels of primary school children and to determine the associations among PF, concentration, and health-related quality of life (HRQOL) in a subcohort. PF was assessed in 6533 healthy primary school-age children (aged 6–10 years, 3248 boys and 3285 girls) via standardized test batteries. Concentration was measured with the d2-R test, and KINDL questionnaires were used to determine HRQOL. Analysis of variance showed an increase in PF with age in all PF dimensions (all *p* < 0.001), except cardiopulmonary fitness (estimated VO2max) in girls (*p* = 0.129). Boys performed better in nearly all PF dimensions, except curl-ups, in all children aged ≥7 years (*p* < 0.05). Concentration levels increased in boys and girls aged 7–9 years (*p* < 0.001), whereas HRQOL did not (*p* = 0.179). The estimated VO2max had a strong impact on concentration (β = 0.16, *p* < 0.001) and HRQOL (β = 0.21, *p* < 0.001) in 9- to 10-year-olds. Cardiopulmonary fitness is important for improved concentration and better HRQOL in primary school-age children. However, longitudinal data are needed to provide further insight into the intraindividual relationships of PF and concentration over the course of child development and set up targeted prevention programs.

## 1. Introduction

Children’s health is influenced by biological, behavioral, and environmental factors [1]. Most research in this field is focused on the impacts of healthy eating, physical activity, sedentary behavior, and sleep [2,3,4,5,6,7]. Recent studies show, that only a few children meet the recommendations of physical activity [8,9,10]. Furthermore, sedentary behavior is increasing in children and adolescents [8].

The health-promoting effect of physical activity in children is widely recognized [11,12,13,14]. Regular exercise improves anthropometric measures like weight and body mass index (BMI) [13,15]. Furthermore, regular physical activity has a positive effect on numerous health outcomes in children and adolescents, including physical fitness (PF), cardiometabolic health, bone health, cognitive abilities (e.g., academic performance), and mental health [2,3,11,14,16,17,18]. This is why recommendations for physical activity are already generated, e.g., by the World Health Organization [11] for children. However, PF is a more objective parameter to quantify when evaluating health promotion, compared with indices of physical activity, such as self-report questionnaires or accelerometers [19]. Furthermore, cardiorespiratory fitness as part of PF is more strongly related to risk factors of cardiovascular disease than physical activity [20].

PF is defined as a person’s ability to perform daily activities with great power, such as endurance, strength, flexibility, coordinative skills, and speed [21], and can be examined using a variety of fitness tests [22,23,24]. Cardiorespiratory fitness is an important health factor that reduces cardiovascular risk, even in children [2,25,26]. Moreover, lower cardiovascular risk in childhood is associated with higher PF in midlife [27].

Children’s health is also influenced by behavioral factors and cognition [1]. Studies reported positive associations between concentration (as a factor of cognition) and PF in children aged 10–12 years [28,29]. Furthermore, a higher PF seems to be linked to better academic performance [29,30]. In the long term, PF in childhood is positively associated with higher concentration levels in adulthood [31]. Another factor influencing children’s health is health-related quality of life (HRQOL) [1]. HRQOL is positively associated with cardiorespiratory fitness and muscular strength in children as early as preschool and in adolescents [32,33,34,35].

In sum, associations among PF, concentration, and quality of life have been demonstrated in adolescents [4,17]. However, concentration and quality of life have not yet been examined in the same population nor in primary school children. These factors might be essential to academic performance and a development-providing environment in school children, especially in age groups younger than 11 years. In the transition phase into adolescence, shifts in concentration have caused physiological, as well as psychological and emotional changes [36]. In consequence, self-esteem and quality of life in adolescence are often lower, when compared with those of younger peers or adults [37].

Therefore, this study aimed to examine the associations among PF, concentration, and quality of life in 6- to 10-year-old children. Additionally, it aimed to determine age and sex differences in these three dimensions to better understand health development in primary school-age children. 

## 2. Materials and Methods

### 2.1. Sample

Data for this cross-sectional study were collected in the Berchtesgaden Area, Germany, from 2016 to 2020, prior to the COVID-19 pandemic. A total of 6533 healthy primary school children (3285 girls, 8.73 ± 1.18 years) from 15 schools were examined by a trained research team during the second quarter of each study year. Students were recruited for the data collection on 1 day for two sports lessons (90 min) at their local sports hall. Written informed consent was obtained from all parents or legal guardians. The study was conducted in line with the tenets of the Declaration of Helsinki and was approved by the local ethics committee (5490/12).

### 2.2. Anthropometrics

Weight was measured to the nearest 100 g using a calibrated digital scale (seca 799, seca, Hamburg, Germany) with the children lightly dressed and without shoes. Height was determined to the closest 0.1 cm with a mobile stadiometer (seca 799, seca), and BMI was calculated via the standard formula. For better interpretation, BMI values were transformed into age and sex-specific German reference percentiles (standardized BMI) and additionally assigned to one of the following weight categories: underweight (BMI < 10th percentile), normal weight (10th ≥ BMI ≤ 90th percentile), overweight (90th > BMI ≤ 97th percentile), and obesity (BMI > 97th percentile) [38]. 

### 2.3. Physical Fitness Assessment

PF was evaluated using the following test battery: push-ups (PU), curl-ups (CU), and standing long jump (SLJ) to determine upper and lower body strength, handgrip strength (HG), and 20-m multistage shuttle run or the Progressive Aerobic Cardiovascular Endurance Run (PACER) to assess health-related fitness. All tests are validated for children [39,40,41].

PU and CU were performed according to FitnessGram^®^ guidelines [23]. CU was performed with knees flexed and feet unanchored. The velocity of PU and CU was set at approximately 1 repetition every 3 s and controlled using an external metronome. The subjects were asked to complete as many repetitions as possible, including one “joker” to a maximum of 100.

SLJ was measured to determine leg power [24]. Students were encouraged to reach two valid attempts, and the best one was used for further evaluation. SLJ had to be performed with both legs, from takeoff to landing. Jumping distance was measured (in cm) from the starting line to the heel of the student.

To determine HG (in kg), students had to sit upright on a chair, with the feet, knees, and hips positioned at 90° angles. A dynamometer (SAEHAN Hydraulic Hand Dynamometer SH5001, SAEHAN Corporation, Masan, Korea) was held with the arm at a right angle and the elbow by the side of the body. The handle of the dynamometer was adjusted to suit the patient’s finger length when required. Subjects had to squeeze the handle for 3 s with maximum effort. Measurements were conducted three times alternately for each side [42]. Only the highest measurement was used for data evaluation. HG values are given absolute (kg) and normalized to body weight (HGrel, kg/kg body weight, arbitrary unit). HGrel is a more accurate parameter when comparing muscular strength between different samples.

Cardiorespiratory fitness was estimated using the PACER test by FitnessGram^®^ [23,39,43]. The 20-m multistage shuttle run requires participants to maintain the pace set by an audio signal, which progressively increases the intensity every minute. The test stops if a participant is not able to reach the relevant marker in time in two consecutive beeps. The raw score from the PACER is the number of laps completed before volitional exhaustion. The total lap number was then used to estimate VO2max following the model 2 equation of Mahar et al. [44].

### 2.4. Concentration

The d2-R test was used to assess concentration and attention capacity [45] and has been previously conducted with school children [46,47]. It is a paper–pencil cancellation test, where subjects have to cross out all “d” letters with two dashes under a time limit. Output variables are the total number of items processed, the total number of errors, and the total performance value [45]. For further evaluation, only the total performance value was used. The d2-R test is conducted on students in the second grade and higher, as younger pupils are not able to complete the questionnaire.

### 2.5. Health-Related Quality of Life

HRQOL was assessed using the KINDL questionnaire [48]. It contains 24 Likert-scaled items, associated with six dimensions: physical well-being, emotional well-being, self-esteem, family, friends, and everyday functioning (=school). The six subscales were combined to calculate the total score, according to guidelines [49]. KINDL was chosen because of its reliability and validity in assessing children [49]. The German kid version of the questionnaire was filled out by students in the fourth grade and higher, as younger pupils are not able to complete the questionnaire without external help.

### 2.6. Data Analysis

Data were analyzed using Microsoft Office Excel (2019, Microsoft, Redmond, Washington, DC, USA) and IBM SPSS Statistics Version 28 (IBM, New York, USA). Group differences (age groups, sex, and weight status) were analyzed using independent samples t- and U-tests and univariate analysis of variance (ANOVA) for PF (*n* = 6533), concentration (*n* = 1387), and HRQOL (*n* = 998), dependent on the number of groups compared. Multiple comparisons were calculated using the Bonferroni model. To determine the associations among PF, HRQOL, and concentration, multiple linear regression analyses were performed in a subcohort of 9- to 10-year-old primary school children (inclusion of beta with 95% confidence intervals (CIs)). The dependent variables were HRQOL and concentration. PF parameters, age, sex, and BMI were set as independent variables. After an initial Pearson correlation analysis, only factors that showed linearity with dependent variables were included in the regression model. All assumptions for multiple linear regression models were met. Bootstrapping with a bias-corrected and accelerated bootstrap (BCa) interval of 2000 samples was performed, and *p* < 0.050 was considered statistically significant.

## 3. Results

### 3.1. Analysis of Descriptive Statistics and Sex Differences

Supplementary Table S1 shows the anthropometric characteristics, PF parameters, concentration ability, and quality of life dimensions of the whole sample and separately for boys and girls. When categorized by BMI percentile, 9%, 79%, 8%, and 5% of school-children were underweight, of normal weight, overweight, and obese, respectively. In the overall sample, 2% more boys were overweight and obese than girls (*p* < 0.001). However, boys showed significantly better scores than their female peers in the following fitness parameters, after controlling for age: PACER distance (number of completed laps, *p* < 0.001), estimated VO2max (*p* < 0.001), HGrel (*p* < 0.001), PU (*p* < 0.001) and SLJ (*p* < 0.001). Conversely, girls scored better on concentration (d2-R total) and HRQOL scores (total, family, and school) (all *p* < 0.05). 

### 3.2. Analysis of Descriptive Statistics and Weight Class Differences

ANOVA showed differences between weight classes in PF (*p* < 0.001 for all), concentration (*p* < 0.033), and all HRQOL scores except family (total, *p* < 0.001; physical well-being, *p* < 0.001; emotional well-being, *p* = 0.010; self-esteem, *p* = 0.027; friends, *p* < 0.007; and school, *p* < 0.001). Overweight and obese children performed significantly worse than children who were underweight or normal-weight on each item of PF (*p* < 0.001). HRQOL total, physical well-being, self-esteem, friends, and school were significantly lower in obese children. No differences between BMI categories on concentration levels were found after post hoc analysis. For more detailed information about pairwise weight class comparisons see Supplementary Figure S2.

### 3.3. Analysis of Descriptive Statistics and Differences between Age Groups in Boys and Girls

Differences between age groups are only considered when they appeared consecutive. Exact post hoc differences between all age groups can be found in Table 1, marked as superscript numbers. ANOVA revealed significant increases in all PF dimensions in boys across all age groups (*p* < 0.001); see Table 1. In girls, only estimated VO_2_ max did not show significant differences between age groups (*p* = 0.129). PACER distance significantly increased in girls in the 7- to 8- and 8- to 9-year age groups (*p* < 0.05). Additionally, 7-year-old boys also achieved a higher PACER distance than their younger peers (*p* < 0.05). Estimated VO_2_ max increased significantly between 7- and 8-, and 9- and 10-year-old boys, whereas cardiopulmonary fitness did not differ among 6- to 10-year-old girls. HG strength significantly increased in both sexes until 8 (boys) and 9 (girls) years. Similar trends could be found for PU. CU and SLJ linearly increased from age 6 to 10 years. Differences in concentration levels were only analyzed among ages 7–10 years. Post hoc differences showed that d2-R increased with age but only showed significant differences between 8- and 9-year-old boys and girls (*p* < 0.05). HRQOL was only assessed in 9- to 10-year-olds, and the total scores did not differ between age groups in either boys or girls. Supplementary Figure S1 shows differences in all parameters among age groups in boys and girls.

### 3.4. Association between Physical Fitness, Concentration, and HRQOL in a Subcohort of 9- to 10- Year-Old Children

**Figure 1:**
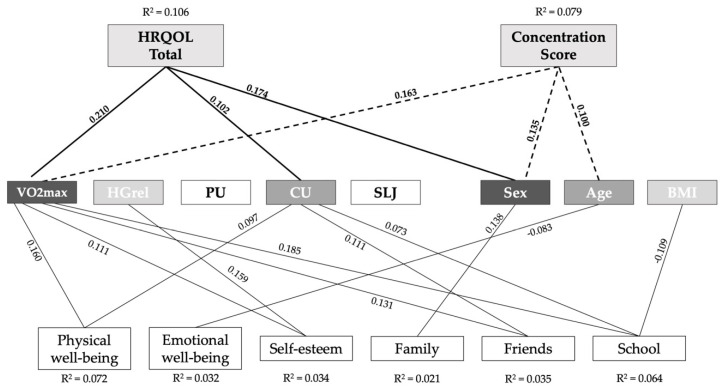
Relationships between HRQOL and concentration at every anthropometric measurement, as well as physical fitness parameters, among 9- to 10-year-olds. From dark gray to white: highest to lowest β in the regression model. Dependent main variables are visualized on the top level, PF parameters and demographic data in the center, and HRQOL subscales on the bottom line. Only significant independent variables are connected to their dependent variables with a black line. The lines of main variables are marked bold.

Higher BMI values were negatively correlated with all PF parameters, concentration, and HRQOL. Higher levels of PF were positively correlated with higher levels of concentration and HRQOL. Exact Pearson correlation results can be found in Supplementary Table S3. The multiple regression model for total HRQOL in 9- to 10-year-old children (*n* = 802; 394 boys and 408 girls) identified sex, estimated VO_2_ max, and CU (adjusted r2 = 0.106; F [7801] = 14.57; *p* < 0.001) as significant factors. All factors used in the model are shown in Table 2. Age was the only factor being eliminated in this regression model. The multiple regression model also showed 10.6% variance was due to HRQOL. The strongest β was found for estimated VO_2_ max (β = 0.21), followed by sex (β = 0.174, positive value for girls) and CU (β = 0.102). Children with a higher estimated VO_2_ max showed higher values in total HRQOL, physical well-being (β = 0.16), self-esteem (β = 0.11), friends (β = 0.13), and school (β = 0.19). Detailed information about all regression models and factors used in the models are shown in Supplementary Table S2. In the multiple linear regression model with concentration as a dependent variable, anthropometric and fitness parameters accounted for 7.8% of the variance (adjusted r^2^ = 0.079, F[7751] = 10.24, *p* < 0.001). Sex (β = 0.18) and estimated VO_2_ max (β = 0.16) were the strongest factors contributing to a higher concentration score, followed by age (β = 0.10). HGrel was rejected in the model. All dependencies identified in the regression models are shown in Figure 1. Dependent main variables are visualized on the top level, PF parameters and demographic data in the center, and HRQOL subscales on the bottom line. Only significant independent variables are connected to their dependent variables with a black line. The lines of main variables are marked bold.

## 4. Discussion

This study sought to investigate the differences in PF, concentration, and HRQOL in boys and girls, as well as between different age groups in primary school. Additionally, we wanted to determine the association among PF, concentration, and HRQOL in 9- to 10-year-old children.

Our main results are as follows:

1. Boys scored significantly better in estimatedVO2max, HG, SLJ, and PU throughout nearly all age groups;
2. Girls showed better concentration scores in the d2-R total score and a better overall HRQOL than boys, independent of age;
3. Estimated VO2max is the strongest predictor for higher concentration levels and a better overall HRQOL.

Caamaño-Navarrete et al. [28] found similar results in fitness levels as we did in our population sample. Chilean boys showed significantly better scores in estimated VO2max, HG, and SLJ than Chilean girls. Fühner et al. [50] confirmed this performance gap in a cohort (*n* = 108,295) of 8-year-old third graders. In all PF tests, including coordination, speed, upper and lower limb power, and endurance, boys outperformed girls. In a German reference cohort, Woll et al. [24] found age to be the dominant influencing parameter of PF. These results are in line with the findings in the present study, wherein age and sex significantly influenced estimated VO2max, PU, CU, SLJ, and HGrel. Data for boys older than 10 years suggested a great increase in endurance performance, whereas girls’ cardiopulmonary fitness seemed to develop linearly until the age of 18 [24]. In our data, estimated VO2max increased until the age of 9 years in both sexes (Supplementary Figure S1), but only boys showed significant changes *(p* < 0.001). Afterward, a decline could already be seen in the 10-year old boys (*p* < 0.001), but not in the girls (*p* > 0.05). Therefore, the developments in the present cohort from the age of 10 years into adolescence would be interesting to observe. HGrel increased with age (*p* < 0.001) and was identified as an important cardiovascular risk marker. In adolescence, higher HG is associated with lower blood pressure values [51]. Furthermore, muscular and cardiorespiratory fitness are inversely correlated with metabolic risk [52].

Contrary to our findings, selective attention and concentration did not interact with PF parameters in the Chilean study [28]. However, in our data, estimated VO2max (β = 0.163, *p* < 0.001) showed a significant association with concentration levels. These contrary findings could be explained by a huge difference in sample size, as Caamaño-Navarrete et al. [28] used the same tests to determine concentration levels, but analyzed a considerably smaller sample size (*n* = 248 vs. *n* = 752). The percentage of variance explained was not extremely strong with a corrected r2 of 0.078. Nevertheless, parts of the model were explained by cardiorespiratory fitness, which means fitter kids can focus better on their given tasks [28]. PF and concentration are also linked to academic performance [29]. McPherson et al. [30] investigated the interactions among physical activity (counted in steps), cognition, and academic performance and found a direct association between physical activity and academic performance with cognition, exhibiting a partial mediating effect (r = 0.225 and r = 0.121). In a cluster analysis, Dumuid et al. [53] found children with unhealthy diet habits and higher screen time to be those with worse academic performance. Therefore, academic performance should be considered as an additional health factor in future studies, as well as screen time and eating patterns.

Only 11% of the variance in HRQOL levels could be explained using our model. Nevertheless, estimated VO2max was the strongest influencing factor in 9- to 10-year-old primary school-age children in multiple HRQOL dimensions (total, physical well-being, friends, and school). Similar results were reported by Evaristo et al. [32] and Andersen et al. [33]. The 20-m shuttle run remained as a mediator for HRQOL in the regression model, whereas muscular fitness (HG and SLJ) lost significance once they were analyzed in the same model [32]. This shows the strong impact of cardiorespiratory fitness on HRQOL in children and adolescents. In our sample, the only model, in which HGrel remained as an explanatory factor was self-esteem (β = 0.16). This is in line with the findings of Bolados et al. [54], who found a positive association between strength and self-esteem in boys. Estimated VO2max was the second strongest factor in our self-esteem model (β = 0.11), which again demonstrates the importance of cardiorespiratory fitness even at a young age. Andersen et al. [33], again, found a significant positive correlation between cardiorespiratory fitness and HRQOL, assessed with the KIDSSCREEN (*p* < 0.050). Authors also found a negative correlation between cardiorespiratory fitness, as well as physical activity, and abdominal adiposity, whereas HG correlated positively. In our analysis, a higher BMI negatively influenced HRQOL only in the school setting (β = −0.11). As BMI as a marker for obesity has been linked to depression in adolescents (12.5 ± 1.1 years) [55], it might also be a sensitive marker for children’s health in the school setting and should therefore always be considered important.

Summarizing our results, cardiorespiratory fitness, as demonstrated through the 20-m shuttle run, is regularly shown to be a holistic indicator for cardiovascular prevention in children and adolescents [56] and should therefore always be considered when testing for PF. As Ortega et al. [21] already showed in their review, school settings are important to promote an active lifestyle. The authors also described that physical fitness programs improved PF independent of chronological age, maturation status, and sex in this age group. As already mentioned, higher PF and higher levels of physical activity led to higher academic performance and a lower cardiovascular risk in the future. Therefore, regular assessments of PF in primary school children are an important tool to detect children with a lower fitness and guide them actively to positive health behaviors. Involving external experts into sport lessons also helps to keep children motivated. This is why more expertise is needed in schools. Therefore, cooperations should be forged between different schools and universities or other preventive stakeholders to assure a continuous monitoring of PF. Additionally, more awareness for the need of an active school setting has to be formed. Regular physical activity and a healthy lifestyle should not only be the educational mission of sports teachers but of all educators working in this field. Nationwide concepts are needed to build a comprehensive PF test battery, bringing together the theoretical background and practical application. These are just a few aspects, to support the worldwide guidelines of preventing non-communicable deseases [57]. First attempts to implement more physical activity in daily school routines have already been made [58], but there is still more research needed in this field, especially in the long term. Furthermore, effects should be monitored to prove the beneficial effect of physical activities and intensive training on cardiovascular and muscular fitness, quality of life, and mental health. As monitoring physical activity is time-intense (via accelerometers) or difficult to assess in children younger than 9 years (via activity questionnaires), we promote annual screenings of PF in a simple and time-saving test battery with the help of external experts to raise motivation in children and adolescents. This approach is especially important, as it would provide a large data set of PF in German children and adolescents and the possibility to create much-needed comparative data.

### Limitations

In total, PF data of 6533 healthy primary school children from 15 schools in the Berchtesgaden area were evaluated. Nearly all primary schools in this area participated in this study. We did not account for sociodemographic parameters in this study, but we assume that our study sample is representative of this selected geographic area, as all children do have to complete primary school in Germany. Therefore, parameters influencing the sociodemographic status, e.g., ethnic backgrounds, religions, educational status, and immigration background are represented in this study cohort. Nevertheless, results cannot be generalized for Bavaria or even Germany. This also becomes clear when analyzing demographic data. BMI values of our sample are lower than German reference values [10]. This might lead to a selection bias, as children with lower BMI values are associated with higher fitness levels [55]. Furthermore, in a recent review, authors found more active children to have lower cardiometabolic risk scores [59].

Additionally, we do have to consider the small r2 values of all regression models. General linear modeling might provide more valuable insights into specific interactions. Nevertheless, our results show, that sex, age and BMI are not always the factors that should be controlled, as they impact models differently (see Table 2 and Supplementary Table S2). In combination with concentration, BMI did not add variance to the regression model (β = −0.18). Total HRQOL was only explained by sex, but not by age and BMI (see Table 2). Additionally, the factors analyzed are only an excerpt of all health determinants. When analyzing PF parameters, age, and sex with the regression models, these are clearly not the only factors influencing concentration and HRQOL. Many other factors should be considered, such as the sociodemographic status of the school children and their overall learning environment. Screen time and sedentary behavior are also important health-promoting factors that should be taken into account [11,59].

Furthermore, in this study, we only analyzed cross-sectional data. To obtain a more in-depth analysis, further studies need to be conducted to observe intraindividual changes over time. Another limitation of this study was that we did not evaluate overall physical performance. Coordination, balance, and flexibility were not included in our study. For a more universal approach, these physical performance factors should also be included in future studies.

## 5. Conclusions

VO2max was found to be one of the main factors influencing concentration levels and HRQOL dimensions in primary school children. PF, especially cardiorespiratory performance, should therefore be promoted more specifically in school settings to support the promotion of an overall healthy lifestyle in children and adolescents. Recommendations on physical activity should also be supplemented with information on PF and its assessment.

## Figures and Tables

**Table 1:** Descriptive statistics and differences between age groups total and differentiated by boys and girls in anthropometric characteristics, physical fitness parameters, concentration, and health-related quality of life.

		Age (Years)										
		6		7		8		9		10		
		*n*	M [CI 95%]	*n*	M [CI 95%]	*n*	M [CI 95%]	*n*	M [CI 95%]	*n*	M [CI 95%]	*p*
**Anthropometrics**												
BMI (kg/m^2^)	Total	552	15.93 [15.76, 16.10] ^8,9,10^	1491	16.16 [16.05, 16.28] ^8,9,10^	1596	16.73 [16.60, 16.85] ^6,7,9,10^	1731	17.23 [17.10, 17.36] ^6,7,8,10^	1143	17.99 [17.80, 18.18] ^6,7,8,9^	<0.001 *
	Girls	291	15.82 [15.57, 16.06] ^8,9,10^	761	16.02 [15.87, 16.17] ^8,9,10^	830	16.67 [16.50, 16.85] ^6,7,9,10^	857	17.13 [16.95, 17.32] ^6,7,8,10^	538	17.76 [17.48, 18.03] ^6,7,8,9^	<0.001 *
	Boys	261	16.06 [15.83, 16.29] ^8,9,10^	730	16.32 [16.15, 16.48] ^8,9,10^	766	16.78 [16.61, 16.96] ^6,7,9,10^	874	17.33 [17.15, 17.51] ^6,7,8,10^	605	18.19 [17.93, 18.45] ^6,7,8,9^	<0.001 *
zBMI	Total	552	0.033 [−0.046, 0.113]	1491	−0.001 [−0.052, 0.050]	1596	0.031 [−0.019, 0.081]	1731	0.030 [−0.018, 0.078]	1143	0.106 [0.043, 0.169]	0.117
	Girls	291	−0.024 [−0.133, 0.085]	761	−0.068 [−0.137, 0.002]	830	−0.003 [−0.073, 0.066]	857	−0.021 [−0.091, 0.050]	538	0.008 [−0.085, 0.100]	0.690
	Boys	261	0.097 [−0.020, 0.213]	730	0.069 [−0.005, 0.143]	766	0.068 [−0.004, 0.140]	874	0.080 [0.014, 0.145]	605	0.194 [0.107, 0.280]	0.145
**Physical Fitness**												
VO2max (ml/kg/min^−1^)	Total	469	44.57 [44.28, 44.86] ^8,9,10^	1312	45.00 [44.77, 45.23] ^8,9^	1409	45.77 [45.49, 46.04] ^6,7^	1549	46.08 [45.80, 46.37] ^6,7,10^	1026	45.47 [45.09, 45.86] ^6,9^	<0.001 *
	Girls	248	44.15 [43.81, 44.48]	670	44.30 [44.05, 44.55]	747	44.77 [44.45, 45.08]	782	44.82 [44.50, 45.15]	476	44.37 [43.92, 44.82]	0.129
	Boys	221	45.05 [44.57, 45.52] ^8,9,10^	642	45.72 [45.34, 46.10] ^8,9^	662	46.90 [46.45, 47.35] ^6,7^	767	47.37 [46.91, 47.82] ^6,7,10^	550	46.43 [45.83, 47.02] ^6,9^	<0.001 *
Pacer (laps)	Total	469	18.57 [17.76, 19.38] ^7,8,9,10^	1312	22.10 [21.45, 22.75] ^6,8,9,10^	1409	27.46 [26.68, 28.24] ^6,7,9,10^	1549	31.47 [30.66, 32.28] ^6,7,8^	1026	32.57 [31.48, 33.66] ^6,7,8^	<0.001 *
	Girls	248	17.35 [16.39, 18.30] ^8,9,10^	670	20.15 [19.43, 20.87] ^8,9,10^	747	24.61 [23.71, 25.50] ^6,7,9,10^	782	27.88 [26.96, 28.81] ^6,7,8^	476	29.43 [28.15, 30.70] ^6,7,8^	<0.001 *
	Boys	221	19.95 [18.62, 21.28] ^7,8,9,10^	642	24.14 [23.05, 25.22] ^6,8,9,10^	662	30.68 [29.40, 31.96] ^6,7,9,10^	767	35.12 [33.83, 36.41] ^6,7,8^	550	35.29 [33.61, 36.97] ^6,7,8^	<0.001 *
HGrel	Total	446	0.416 [0.405, 0.428] ^7,8,9,10^	1215	0.452 [0.446, 0.459] ^6,8,9,10^	1342	0.481 [0.475, 0.487] ^6,7,9,10^	1421	0.499 [0.493, 0.504] ^6,7,8^	934	0.500 [0.493, 0.507] ^6,7,8^	<0.001 *
	Girls	231	0.390 [0.374, 0.406] ^7,8,9,10^	617	0.434 [0.425, 0.443] ^6,8,9,10^	696	0.458 [0.450, 0.467] ^6,7,9,10^	698	0.477 [0.469, 0.485] ^6,7,8^	444	0.482 [0.472, 0.492] ^6,7,8^	<0.001 *
	Boys	215	0.445 [0.429, 0.461] ^7,8,9,10^	598	0.471 [0.461, 0.480] ^6,8,9,10^	646	0.506 [0.497, 0.515] ^6,7^	723	0.519 [0.511, 0.528] ^6,7^	490	0.516 [0.506, 0.526] ^6,7^	<0.001 *
PU (number)	Total	549	6.82 [6.17, 7.47] ^7,8,9,10^	1481	8.85 [8.39, 9.31] ^6,8,9,10^	1570	10.95 [10.46, 11.43] ^6,7,9^	1710	12.01 [11.51, 12.52] ^6,7,8^	111	11.97 [11.36, 12.59] ^6,7^	<0.001 *
	Girls	291	5.53 [4.76, 6.30] ^7,8,9,10^	751	7.57 [7.00, 8.14] ^6,9,10^	813	8.59 [8.03, 9.15] ^6,10^	845	9.75 [9.16, 10.35] ^6,7^	523	10.14 [9.35, 10.92] ^6,7,8^	<0.001 *
	Boys	258	8.28 [7.23, 9.33] ^8,9,10^	730	10.17 [9.46, 10.88] ^8,,9,10^	757	13.47 [12.71, 14.24] ^6,7^	865	14.23 [13.44, 15.01] ^6,7^	588	13.60 [12.69, 14.52] ^6,7^	<0.001 *
CU (number)	Total	550	10.92 [9.88, 11.97] ^7,8,9,10^	1489	16.69 [15.86, 17.53] ^6,8,9,10^	1582	27.89 [26.64, 29.14] ^6,7,9,10^	1725	36.01 [34.63, 37.39] ^6,7,8,10^	1132	39.55 [37.77, 41.34] ^6,7,8,9^	<0.001 *
	Girls	290	11.00 [9.72, 12.28] ^7,8,9,10^	757	18.07 [16.85, 19.29] ^6,8,9,10^	822	27.94 [26.22, 29.65] ^6,7,9,10^	851	36.95 [34.94, 38.95] ^6,7,8^	535	39.83 [37.29, 42.38] ^6,7,8^	<0.001 *
	Boys	260	10.84 [9.14, 12.53] ^8,9,10^	732	15.27 [14.15, 16.39] ^8,9,10^	760	27.85 [26.01, 29.68] ^6,7,9,10^	874	35.10 [33.20, 36.99] ^6,7,8,10^	597	39.30 [36.80, 41.81] ^6,7,8,9^	<0.001 *
SLJ (cm)	Total	550	107.2 [105.7, 108.7] ^7,8,9,10^	1491	115.1 [114.1, 116.0] ^6,8,9,10^	1585	124.1 [123.1, 125.1] ^6,7,9,10^	1728	132.8 [131.8, 133.8] ^6,7,8,10^	1136	137.1 [135.8, 138.4] ^6,7,8,9^	<0.001 *
	Girls	290	103.8 [102.0, 105.7] ^7,8,9,10^	758	111.6 [110.3, 112.9] ^6,8,9,10^	824	120.1 [118.8, 121.4] ^6,7,9,10^	857	129.2 [127.9, 130.6] ^6,7,8,10^	532	133.9 [132.1, 135.6] ^6,7,8,9^	<0.001 *
	Boys	260	111.0 [108.6, 113.3] ^7,8,9,10^	733	118.6 [117.3, 120.0] ^6,8,9,10^	761	128.5 [127.0, 130.0] ^6,7,9,10^	871	136.3 [134.9, 137.8] ^6,7,8,10^	604	140.0 [138.1, 141.8] ^6,7,8,9^	<0.001 *
**Concentration**												
d2-R (score)	Total			123	72.50 [69.54, 75.45] ^8,9,10^	414	79.67 [77.52, 81.81] ^7,9,10^	508	94.19 [92.38, 96.00] ^7,8^	342	98.23 [95.56, 100.89] ^7,8^	<0.001 *
	Girls			67	73.90 [69.60, 78.19] ^9,10^	218	80.13 [77.02, 83.24] ^9,10^	257	96.99 [94.47, 99.51] ^7,8^	162	100.75 [96.76, 104.73] ^7,8^	<0.001 *
	Boys			56	70.82 [66.74, 74.90] ^9,10^	196	79.15 [76.19, 82.11] ^9,10^	251	91.32 [88.75, 93.90] ^7,8^	180	95.96 [92.37, 99.54] ^7,8^	<0.001 *
**HRQOL**												
HRQOL Total (score)	Total							326	76.73 [75.63, 77.83]	672	75.78 [74.98, 76.58]	0.179
	Girls							176	77.06 [75.68, 78.44]	339	77.08 [75.97, 78.19]	0.987
	Boys							150	76.34 [74.55, 78.12]	333	74.46 [73.31, 75.61]	0.066
Physical well-being (score)	Total							326	79.52 [77.87, 81.18]	672	79.00 [77.82, 80.17]	0.612
	Girls							176	79.73 [77.59, 81.88]	339	78.60 [76.92, 80.28]	0.429
	Boys							150	79.28 [76.70, 81.86]	333	79.40 [77.74, 81.06]	0.937
Emotional well-being (score)	Total							326	83.94 [82.66, 85.22] ^10^	672	82.17 [81.11, 83.22] ^9^	0.036 *
	Girls							176	83.53 [81.89, 85.18]	339	83.14 [81.71, 84.58]	0.751
	Boys							150	84.42 [82.40, 86.44] ^10^	333	81.17 [79.62, 82.73] ^9^	0.013 *
Self-esteem (score)	Total							326	61.73 [59.48, 63.99]	672	61.61 [60.07, 63.15]	0.929
	Girls							176	61.49 [58.74, 64.25]	339	62.86 [60.75, 64.97]	0.474
	Boys							150	62.01 [58.28, 65.75]	333	60.34 [58.09, 62.60]	0.406
Family (score)	Total							326	84.33 [82.63, 86.03]	672	84.09 [82.88, 85.29]	0.819
	Girls							176	86.62 [84.83, 88.41]	339	86.20 [84.68, 87.71]	0.769
	Boys							150	81.64 [78.64, 84.64]	333	81.94 [80.09, 83.79]	0.846
Friends (score)	Total							326	76.83 [75.02, 78.65]	672	77.24 [75.95, 78.52]	0.722
	Girls							176	76.66 [74.22, 79.09]	339	78.88 [77.11, 80.64]	0.155
	Boys							150	77.04 [74.30, 79.78]	333	75.57 [73.71, 77.43]	0.373
School (score)	Total							326	73.84 [71.99, 75.70] ^10^	672	70.50 [69.09, 71.90] ^9^	0.005 *
	Girls							176	74.11 [71.61, 76.61]	339	72.62 [70.65, 74.58]	0.372
	Boys							150	73.53 [70.73, 76.33] ^10^	333	68.34 [66.33, 70.34] ^9^	0.003 *

Values are expressed as number or mean [95% CI]. BMI = body mass index, zBMI = standardized BMI, HGrel = handgrip strength relativized to body weight, PU = push-ups, CU = curl-ups, SLJ = standing long jump, d2-R = concentration test, HRQOL = health-related quality of life, CI = confidence interval; * *p* < 0.05; ^6,7,8,9,10^ = significant post hoc differences between age groups.

**Table 2:** Multiple linear regression analyses for concentration, HRQOL, and physical fitness parameters in 9- to 10-year-old children.

Coefficients	*b* [95% CI_b_] ^a^	Se_b_^a^	β	*t*	*p*
Dependent variable: Total HRQOL (*n* = 802)					
Sex	3.661 [2.259, 5.102]	0.76	0.17	4904	<0.001 *
BMI	−0.005 [−0.282, 0.295]	0.14	−0.00	−0.033	0.973
VO2max	0.340 [0.191, 0.488]	0.07	0.21	4691	<0.001 *
HGrel	4.265 [−3.477, 11.98]	4.12	0.04	1073	0.284
PU	0.073 [−0.008, 0.150]	0.04	0.07	1710	0.088
CU	0.033 [0.012, 0.055]	0.01	0.10	2828	0.005 *
SLJ	0.003 [−0.041, 0.050]	0.02	0.01	0.154	0.878
Dependent variable: Concentration (*n* = 752)					
Age	4.522 [1.127, 7.653]	1.76	0.10	2.779	0.006 *
Sex	7.909 [4.752, 11.36]	1.74	0.18	4.743	<0.001 *
BMI	0.112 [−0.520, 0.744]	0.32	0.01	0.348	0.728
VO2max	0.594 [0.260, 0.927]	0.16	0.16	3.494	<0.001 *
PU	0.127 [−0.066, 0.321]	0.09	0.06	1.290	0.198
CU	0.013 [−0.038, 0.064]	0.02	0.02	0.507	0.612
SLJ	0.087 [−0.006, 0.179]	0.04	0.08	1.841	0.066

^a^ Se_b_ = Confidence interval and Coefficients Standard Error per BCa-Bootstrapping with 2000 BCa samples; *b* = unstandardized beta coefficient, β = standardized beta coefficient, BMI = body mass index, HGrel = handgrip strength relativized to body mass, PU = Push-Ups, CU = Curl-Ups, SLJ = Standing long jump, HRQOL total = KINDL total score, * *p* < 0.05.

## Data Availability

The data is available upon request from the corresponding author.

## Supplementary Materials

The following are available online at https://www.mdpi.com/article/10.3390/jcm11051326/s1, Figure S1: Differences between boys and girls in all parameters, Figure S2: Differences between weight classes in all parameters, Table S1: Descriptive statistics and differences between age groups total and differentiated by boys and girls in anthropometric characteristics, physical fitness parameters, concentration and health-related quality of life, Table S2: Multiple linear regression analyses between HRQOL subscales and physical fitness parameters. Table S3: Correlation coefficients of all PF parameters, concentration score, and all HRQOL scores.
