# Supplementary material for: A Better Cardiopulmonary Fitness Is Associated with Improved Concentration Level and Health-Related Quality of Life in Primary School Children
Source: J Clin Med. 2022 Feb 28;11(5):1326. doi: 10.3390/jcm11051326 (PMC8911456; doi:10.3390/jcm11051326)
Supplement: Supplementary file 1 [file jcm-11-01326-s001.zip › jcm-1510622-supplementary/Supplementary_new/Supplementary Table S3.pdf]

Supplementary Table S3. Correlation coefficients of all PF parameters, concentration score and all HRQOL scores

	M	SD	Age	BMI	VO2max	Pacer	HGrel	PU	CU	SLJ	d2-R	HRQOL total	Physical well-being	Emotional well-being	Self-esteem	Family	Friends	School
**Age**	9.85	0.51	--<br>2887															
**BMI**	17.53	2.97	.136**<br>[.100, .172]<br>2874	--<br>2874														
**VO2max**	45.84	5.97	-.049*<br>[-.087, -.010]<br>2575	-.388**<br>[-.420, -.354]<br>2567	--<br>2575													
**Pacer**	31.91	16.91	.048*<br>[.009, .086]<br>2575	-.374**<br>[-.407, -.340]<br>2567	.995**<br>[.995, .996]<br>2575	--<br>2575												
**HGrel**	0.499	0.113	.024<br>[-.017, .064]<br>2355	-.440**<br>[-.472, -.407]<br>2355	.403**<br>[.366, .438]<br>2122	.405**<br>[.368, .440]<br>2122	--<br>2355											
**PU**	12.00	10.56	.001<br>[-.036, .038]<br>2821	-.281**<br>[-.315, -.246]<br>2813	.546**<br>[.518, .573]<br>2525	-.545**<br>[.517, .572]<br>2525	.435**<br>[.401, .467]<br>2311	--<br>2821										
**CU**	37.41	29.79	.076**<br>[.040, .113]<br>2857	-.214**<br>[-.248, -.178]<br>2849	.282**<br>[.246, .317]<br>2556	-.290**<br>[.254, .325]<br>2556	.166**<br>[.127, .206]<br>2338	.238**<br>[.203, .273]<br>2810	--<br>2857									
**SLJ**	134.52	21.66	.119**<br>[.082, .155]<br>2864	-.311**<br>[-.344, -.278]<br>2855	.537**<br>[.508, .563]<br>2568	.547**<br>[.520, .574]<br>2568	.501**<br>[.469, .530]<br>2342	.496**<br>[.467, .523]<br>2808	.325**<br>[.292, .358]<br>2839	--<br>2864								
**d2-R**	95.81	22.68	.089**<br>[.022, .155]<br>850	-.108**<br>[-.174, -041]<br>845	.225**<br>[.157, .291]<br>771	.232**<br>[.164, .298]<br>771	-.002<br>[-.072, .068]<br>786	.135**<br>[.068, .202]<br>830	.119**<br>[.051, .185]<br>837	.202**<br>[.137, .266]<br>844	--<br>850							
**HRQOL total**	76.09	10.45	-.063*<br>[-.125, -.001]<br>998	-.159**<br>[-.219, -.098]<br>991	.247**<br>[.185, .307]<br>913	.244**<br>[.182, .304]<br>913	.134**<br>[.069, .198]<br>888	.179**<br>[.118, .239]<br>976	.181**<br>[.120, .241]<br>988	.183**<br>[.122, .242]<br>988	.128**<br>[.033, .221]<br>422	--<br>998						
**Physical well-being**	79.17	15.42	-.034<br>[-.096, .028]<br>998	-.113**<br>[-.174, -.051]<br>991	.245**<br>[.183, .305]<br>913	.243**<br>[.181, .303]<br>913	.125**<br>[.060, .190]<br>888	.198**<br>[.137, .257]<br>976	.164**<br>[.103, .224]<br>988	.201**<br>[.140, .260]<br>988	.114*<br>[.018, .207]<br>422	.575**<br>[.532, .615]<br>998	--<br>998					
**Emotional well-being**	82.75	13.30	-.069*<br>[-.130, -.007]<br>998	-.100**<br>[-.161, -.038]<br>991	.150**<br>[.086, .213]<br>913	.146**<br>[.082, .209]<br>913	.061<br>[-.005, .126]<br>888	.123**<br>[.061, .184]<br>976	.091**<br>[.029, .153]<br>988	.126**<br>[.064, .186]<br>988	.142**<br>[.047, .234]<br>422	.655**<br>[.618, .689]<br>998	.383**<br>[.328, .434]<br>998	--<br>998				
**Self-esteem**	61.65	20.47	-.019<br>[-.081, .043]<br>998	-.108**<br>[-.169, -.046]<br>991	.148**<br>[.084, .211]<br>913	.148**<br>[.084, .211]<br>913	.153**<br>[.088, .217]<br>888	.104**<br>[.042, .166]<br>976	.096**<br>[.034, .158]<br>988	.117**<br>[.055, .178]<br>988	.025<br>[-.071, .120]<br>422	.632**<br>[.593, .667]<br>998	.244**<br>[.184, .301]<br>998	.275**<br>[.217, .332]<br>998	--<br>998			
**Family**	84.17	15.77	-.030<br>[-.092, .032]<br>998	-.021<br>[-.083, .042]<br>991	.013<br>[-.052, .078]<br>913	.011<br>[-.054, .076]<br>913	.015<br>[-.051, .080]<br>888	.024<br>[-.039, .086]<br>976	.056<br>[-.006, .118]<br>988	.022<br>[-.041, .084]<br>988	.053<br>[-.043, .148]<br>422	.579**<br>[.536, .619]<br>998	.154**<br>[.093, .214]<br>998	.277**<br>[.219, .333]<br>998	.186**<br>[.126, .246]<br>998	--<br>998		
**Friends**	77.11	16.85	.000<br>[-.062, .062]<br>998	-.084**<br>[-.146, -.022]<br>991	.167**<br>[.103, .229]<br>913	.166**<br>[.103, .229]<br>913	.056<br>[-.010, .121]<br>888	.094**<br>[.031, .156]<br>976	.141**<br>[.080, .202]<br>988	.110**<br>[.048, .171]<br>988	.013<br>[-.082, .109]<br>422	.665**<br>[.629, .698]<br>998	.225**<br>[.166, .283]<br>998	.375**<br>[.320, .427]<br>998	.286**<br>[.228, .342]<br>998	.301**<br>[.243, .356]<br>998	--<br>998	
**School**	71.59	18.16	-.089**<br>[-.150, -.027]<br>998	-.162**<br>[-.222, -.101]<br>991	.212**<br>[.149, .273]<br>913	.207**<br>[.144, .269]<br>913	.079*<br>[.014, .144]<br>888	.137**<br>[.075, .198]<br>976	.129**<br>[.067, .190]<br>988	.116**<br>[.054, .177]<br>988	.153**<br>[.058, .245]<br>422	.649**<br>[.611, .683]<br>998	.244**<br>[.184, .301]<br>998	.308**<br>[.251, .363]<br>998	.218**<br>[.158, .277]<br>998	.307**<br>[.250, .362]<br>998	.316**<br>[.258, .370]<br>998	--<br>998

Data are given as *r* [CI 95%] n; BMI = body mass index, HGrel = handgrip strength relativized to body weight, PU = Push-Ups, CU = Curl-Ups, SLJ = Standing long jump, d2-R = concentration test, HRQOL = health-related quality of life; * $p < .05$, ** $p < .01$
