# Supplementary material for: A Better Cardiopulmonary Fitness Is Associated with Improved Concentration Level and Health-Related Quality of Life in Primary School Children
Source: J Clin Med. 2022 Feb 28;11(5):1326. doi: 10.3390/jcm11051326 (PMC8911456; doi:10.3390/jcm11051326)
Supplement: Supplementary file 1 [file jcm-11-01326-s001.zip › jcm-1510622-supplementary/Supplementary_new/Supplementary_Figure_S1.pdf]

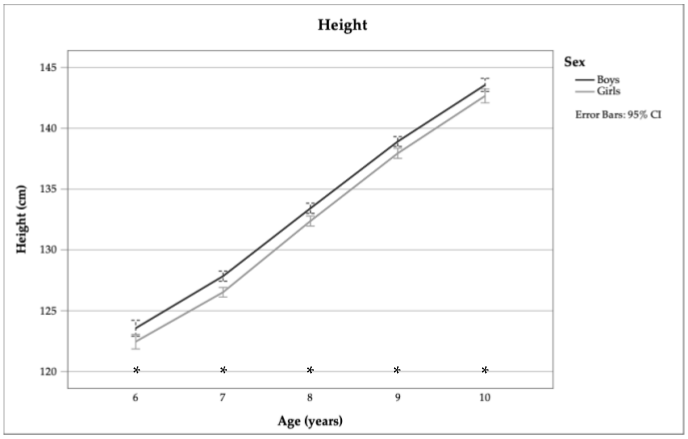
Height
Height (cm)
145
140
135
130
125
120
6
7
8
9
10
Age (years)
Sex
Boys
Girls
Error Bars: 95% CI
*
*
*
*
*

(a)

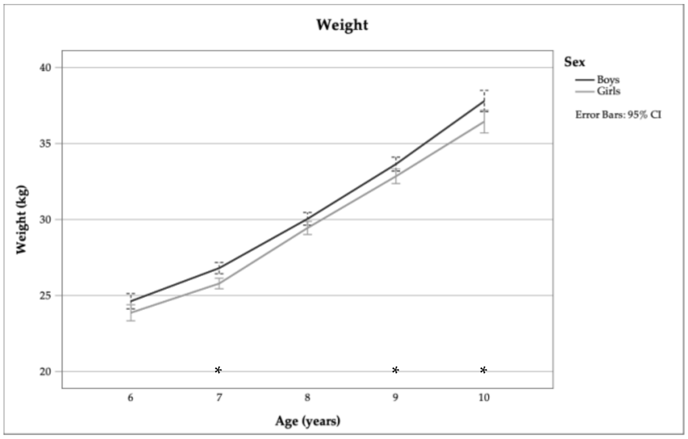
Weight
Weight (kg)
40
35
30
25
20
6
7
8
9
10
Age (years)
Sex
Boys
Girls
Error Bars: 95% CI
*
*
*

(b)

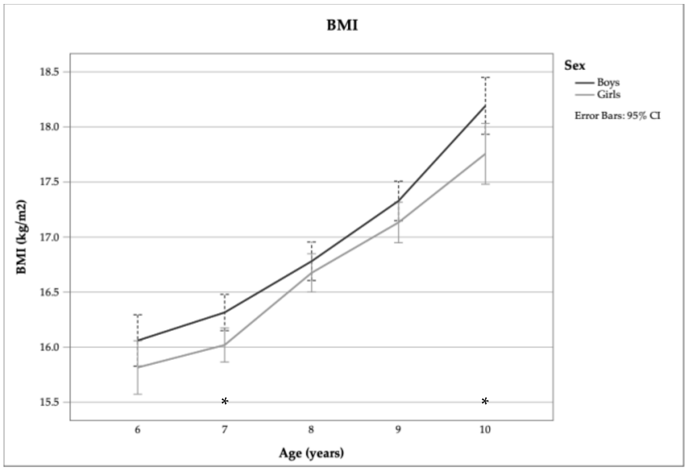
BMI
BMI (kg/m2)
18.5
18.0
17.5
17.0
16.5
16.0
15.5
6
7
8
9
10
Age (years)
Sex
Boys
Girls
Error Bars: 95% CI
*
*

(c)

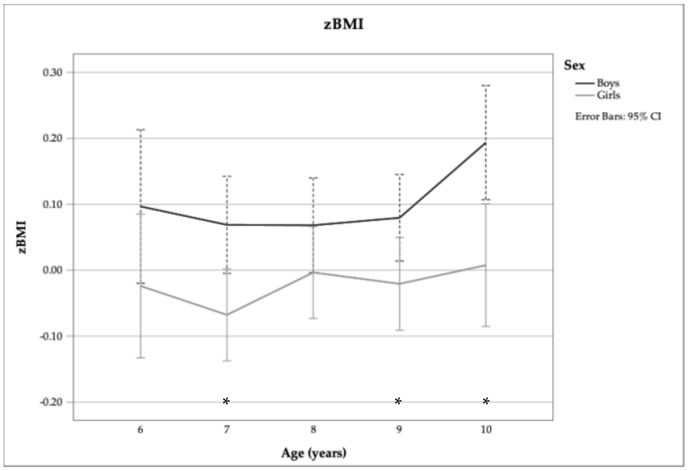
zBMI
zBMI
0.30
0.20
0.10
0.00
-0.10
-0.20
6
7
8
9
10
Age (years)
Sex
Boys
Girls
Error Bars: 95% CI
*
*
*

(d)

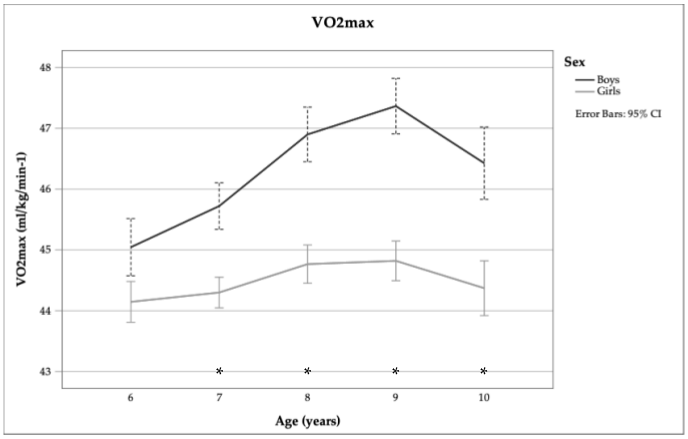
VO2max
VO2max (ml/kg/min-1)
48
47
46
45
44
43
6
7
8
9
10
Age (years)
Sex
Boys
Girls
Error Bars: 95% CI
*
*
*
*

(e)

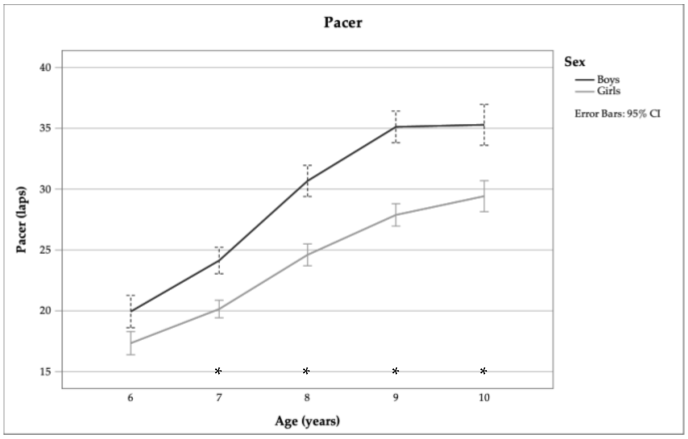
Pacer
Pacer (laps)
40
35
30
25
20
15
6
7
8
9
10
Age (years)
Sex
Boys
Girls
Error Bars: 95% CI
*
*
*
*

(f)

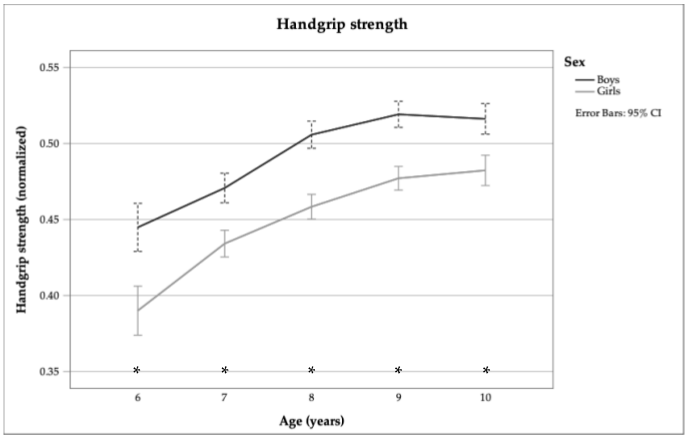
Handgrip strength
Handgrip strength (normalized)
0.55
0.50
0.45
0.40
0.35
6
7
8
9
10
Age (years)
Sex
Boys
Girls
Error Bars: 95% CI
* * * * *

(g)

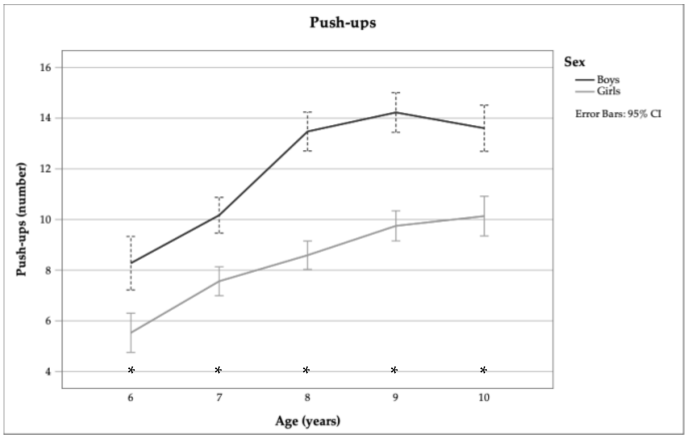
Push-ups
Push-ups (number)
16
14
12
10
8
6
4
6
7
8
9
10
Age (years)
Sex
Boys
Girls
Error Bars: 95% CI
* * * * *

(h)

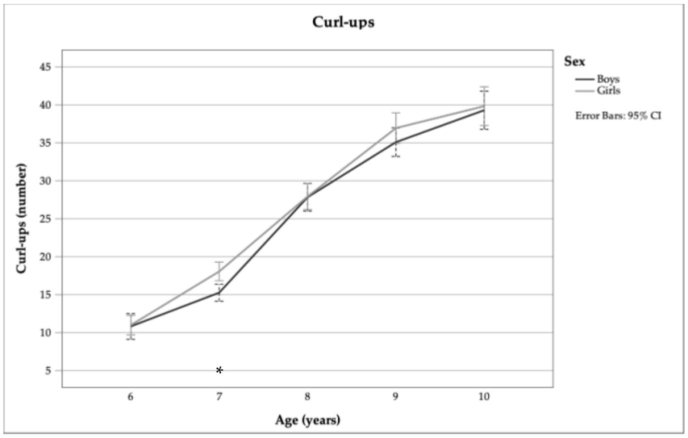
Curl-ups
Curl-ups (number)
45
40
35
30
25
20
15
10
5
6
7
8
9
10
Age (years)
Sex
Boys
Girls
Error Bars: 95% CI
*

(i)

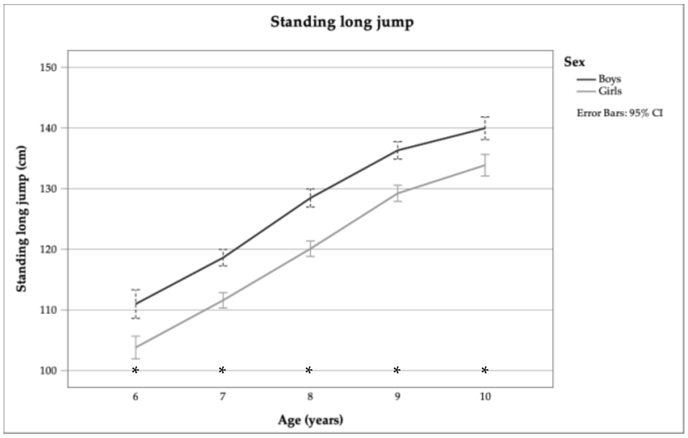
Standing long jump
Standing long jump (cm)
150
140
130
120
110
100
6
7
8
9
10
Age (years)
Sex
Boys
Girls
Error Bars: 95% CI
* * * * *

(j)

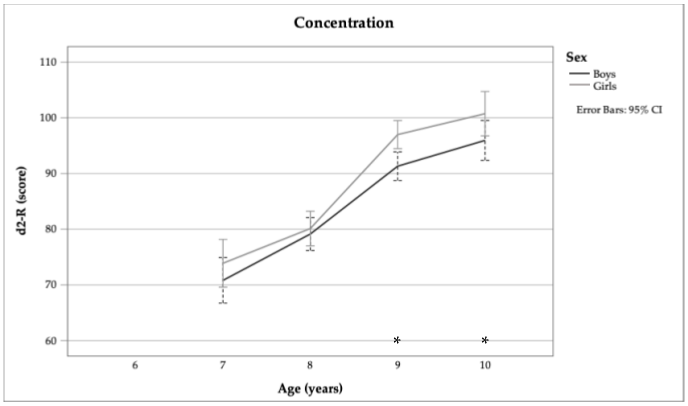
Concentration
d2-R (score)
110
100
90
80
70
60
6
7
8
9
10
Age (years)
Sex
Boys
Girls
Error Bars: 95% CI
* *

(k)

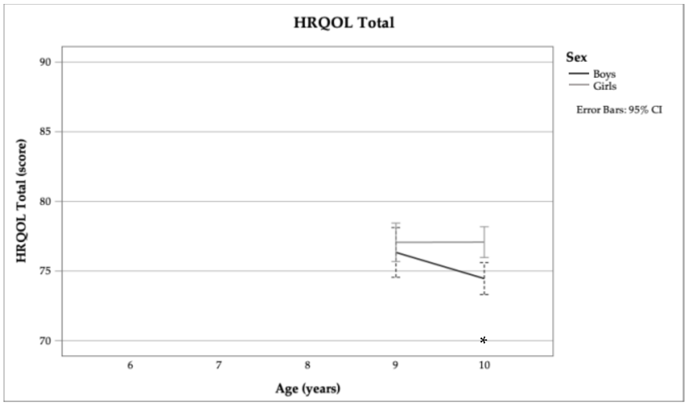
HRQOL Total
HRQOL Total (score)
90
85
80
75
70
6
7
8
9
10
Age (years)
Sex
Boys
Girls
Error Bars: 95% CI
*

(l)

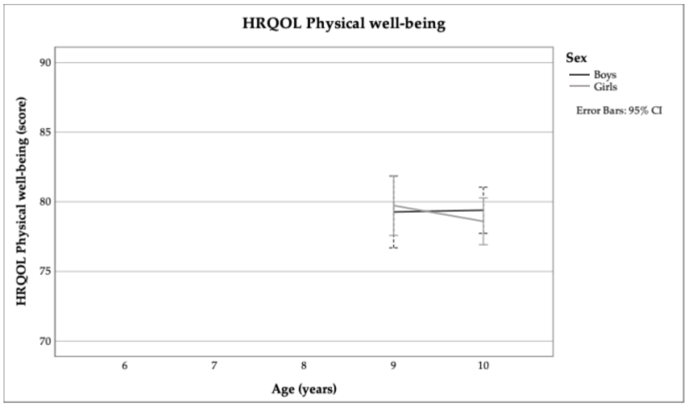

(m)

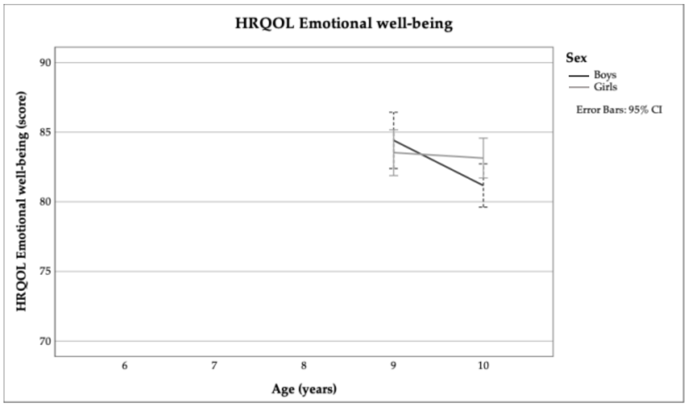

(n)

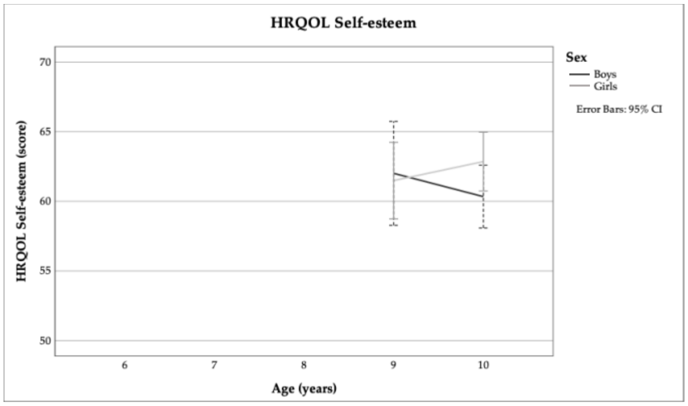

(o)

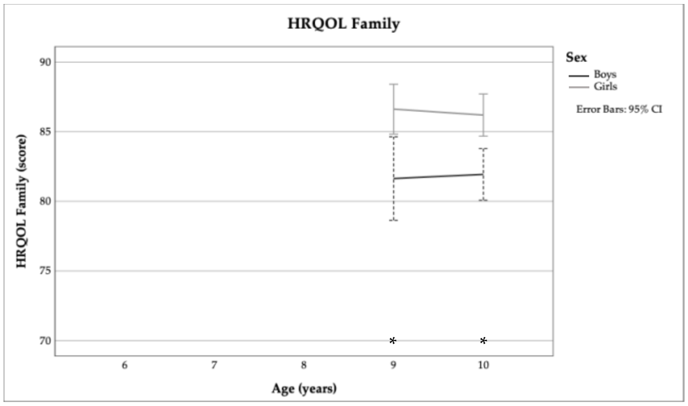

(p)

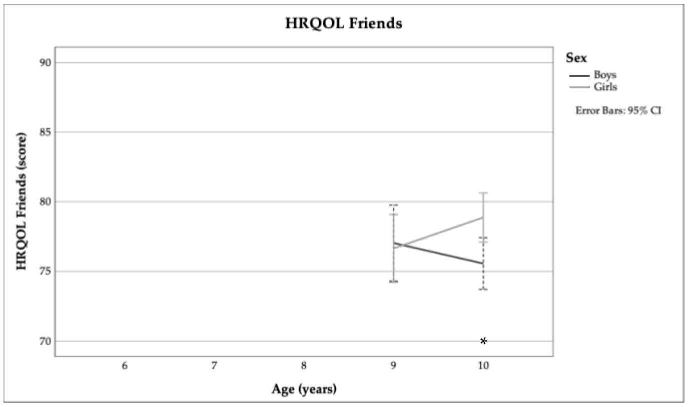

(q)

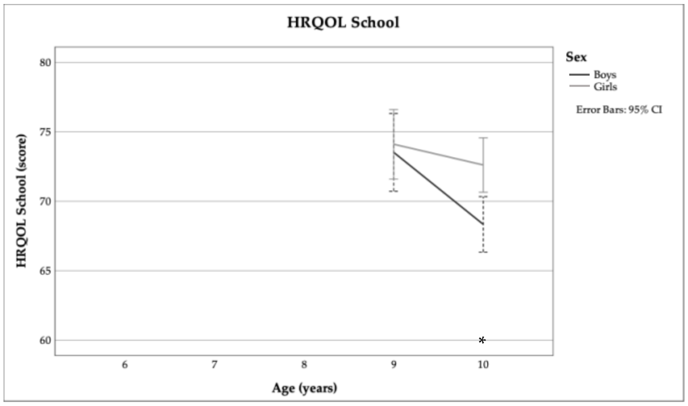

(r)

Supplementary Figure S1. Differences between boys and girls in all parameters, (a)-(d) anthropometrics, (e) – (j) physical fitness tests, (k) concentration score, (l) – (r) health-related Quality of Life (HRQOL) sub-scales
