# Supplementary material for: A Better Cardiopulmonary Fitness Is Associated with Improved Concentration Level and Health-Related Quality of Life in Primary School Children
Source: J Clin Med. 2022 Feb 28;11(5):1326. doi: 10.3390/jcm11051326 (PMC8911456; doi:10.3390/jcm11051326)
Supplement: Supplementary file 1 [file jcm-11-01326-s001.zip › jcm-1510622-supplementary/Supplementary_new/Supplementary_Figure_S2.pdf]

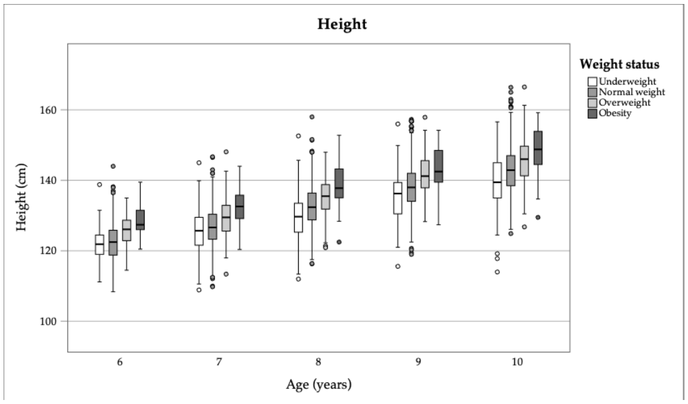
Height
Weight status
Underweight
Normal weight
Overweight
Obesity
Height (cm)
100
120
140
160
6
7
8
9
10
Age (years)

(a)

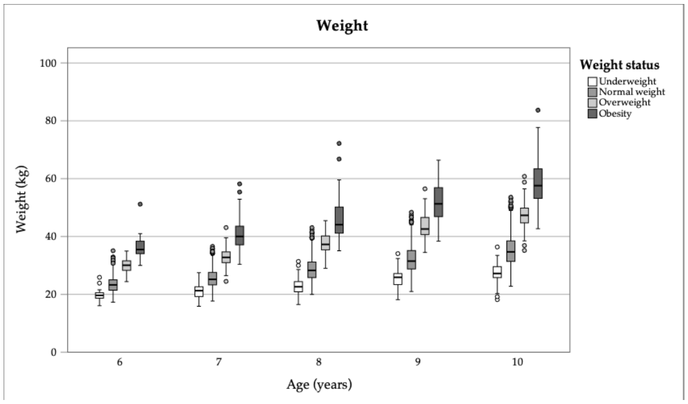
Weight
Weight status
Underweight
Normal weight
Overweight
Obesity
Weight (kg)
0
20
40
60
80
100
6
7
8
9
10
Age (years)

(b)

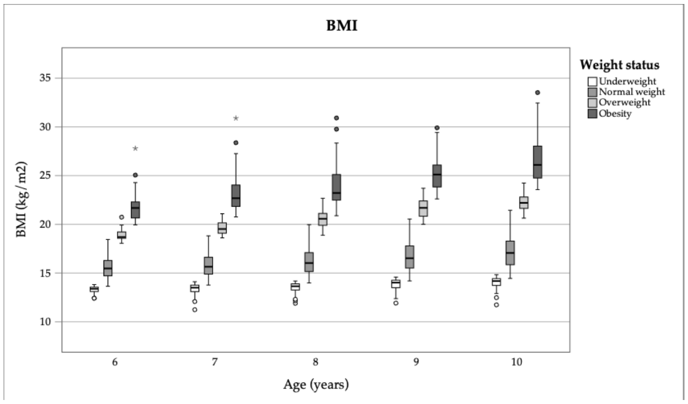
BMI
Weight status
Underweight
Normal weight
Overweight
Obesity
BMI (kg/m2)
10
15
20
25
30
35
6
7
8
9
10
Age (years)

(c)

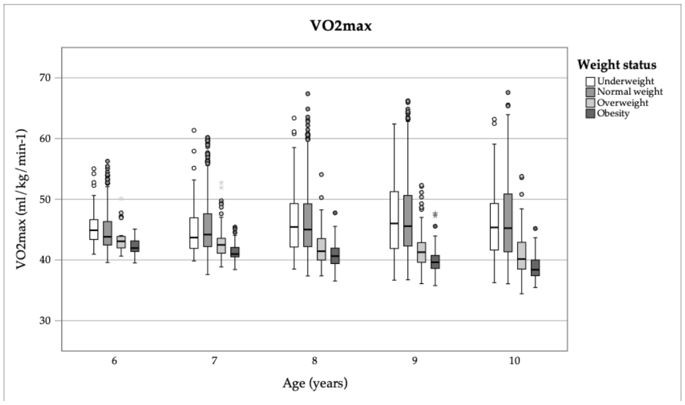
VO2max
Weight status
Underweight
Normal weight
Overweight
Obesity
VO2max (ml/kg/min-1)
30
40
50
60
70
6
7
8
9
10
Age (years)

(d)

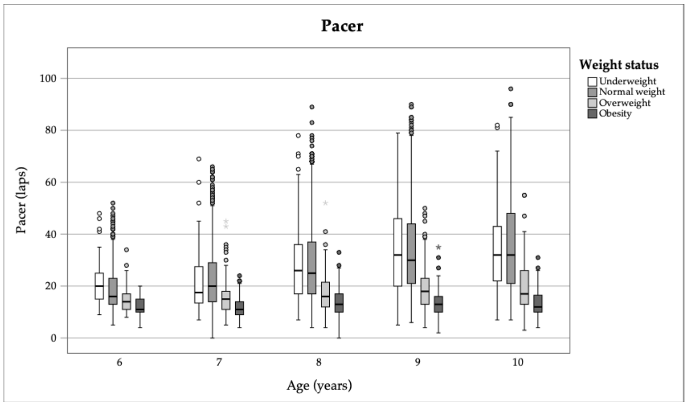
Pacer
Weight status
Underweight
Normal weight
Overweight
Obesity
Pacer (laps)
0
20
40
60
80
100
6
7
8
9
10
Age (years)

(e)

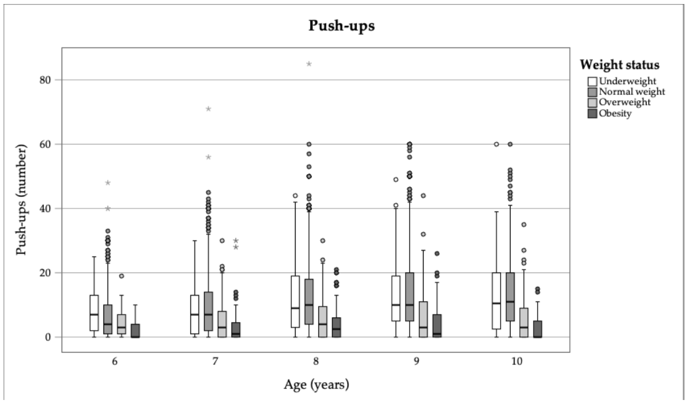
Push-ups
Weight status
Underweight
Normal weight
Overweight
Obesity
Push-ups (number)
0
20
40
60
80
6
7
8
9
10
Age (years)

(f)

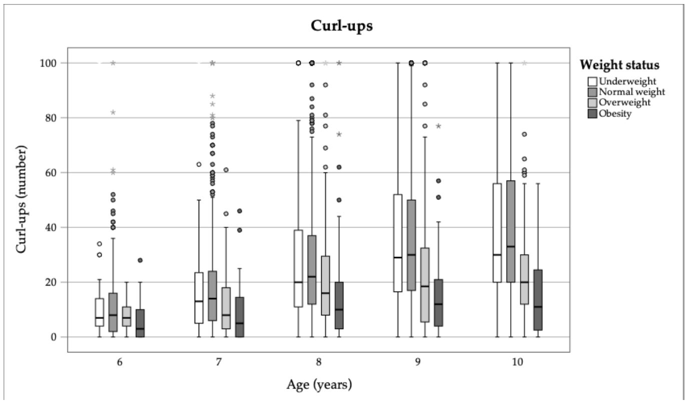
Curl-ups
Curl-ups (number)
Age (years)
Weight status
Underweight
Normal weight
Overweight
Obesity

(g)

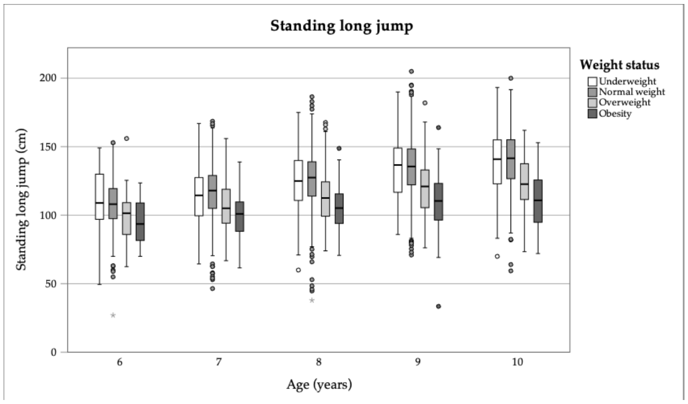
Standing long jump
Standing long jump (cm)
Age (years)
Weight status
Underweight
Normal weight
Overweight
Obesity

(h)

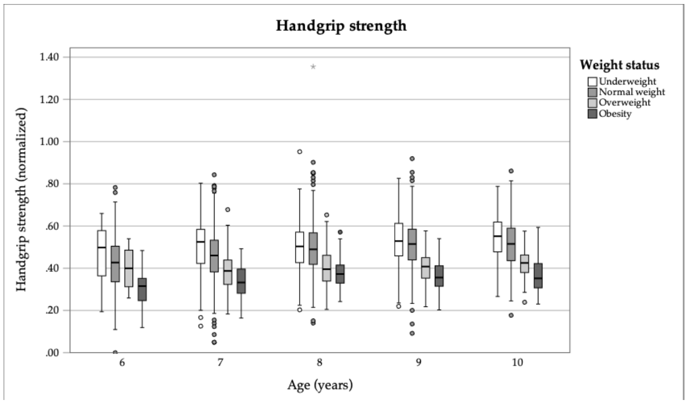
Handgrip strength
Handgrip strength (normalized)
Age (years)
Weight status
Underweight
Normal weight
Overweight
Obesity

(i)

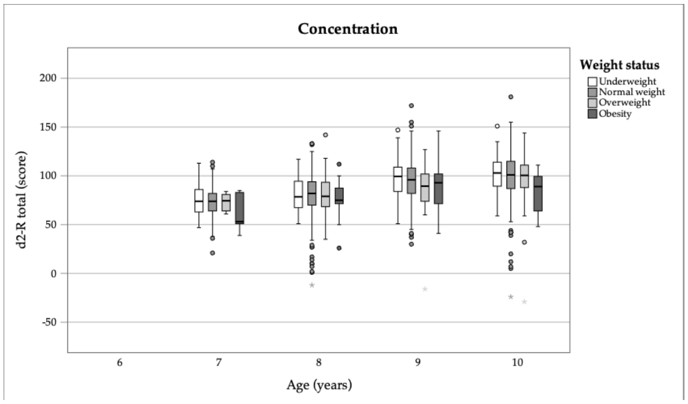
Concentration
d2-R total (score)
Age (years)
Weight status
Underweight
Normal weight
Overweight
Obesity

(j)

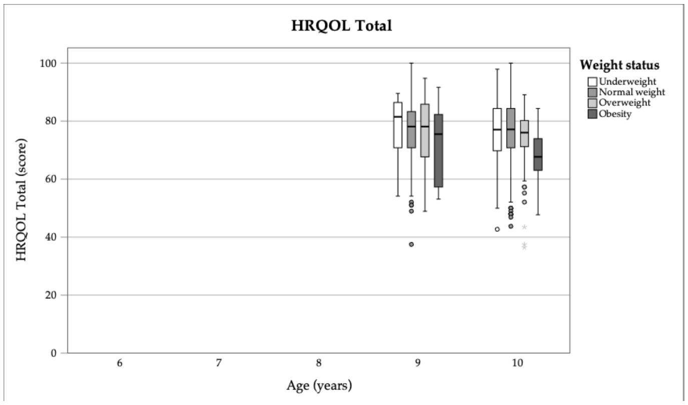
HRQOL Total
HRQOL Total (score)
Age (years)
Weight status
Underweight
Normal weight
Overweight
Obesity

(k)

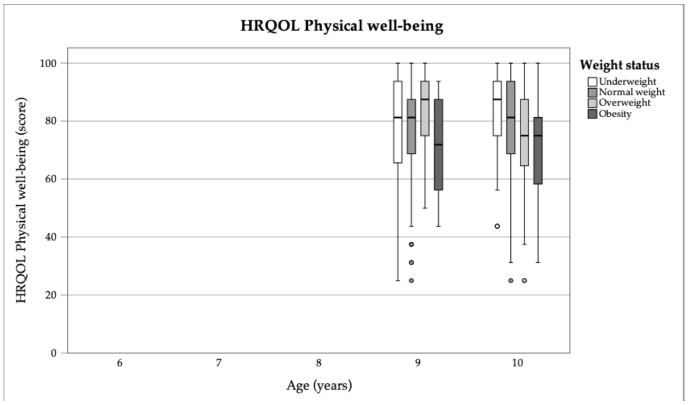
HRQOL Physical well-being
HRQOL Physical well-being (score)
Age (years)
Weight status
Underweight
Normal weight
Overweight
Obesity

(l)

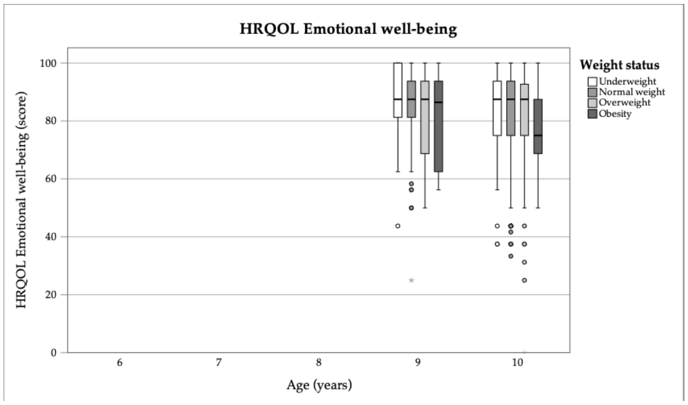


(m)

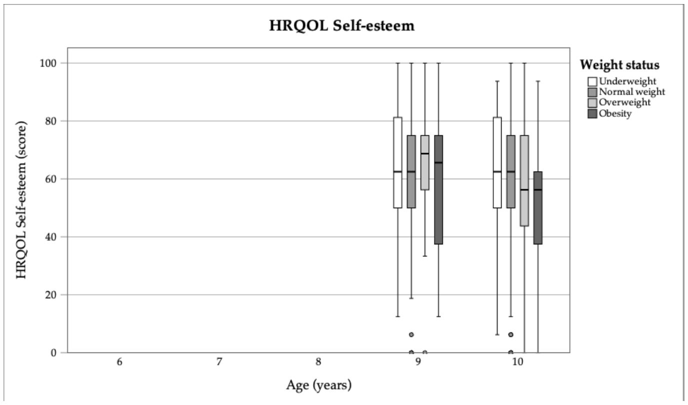


(n)

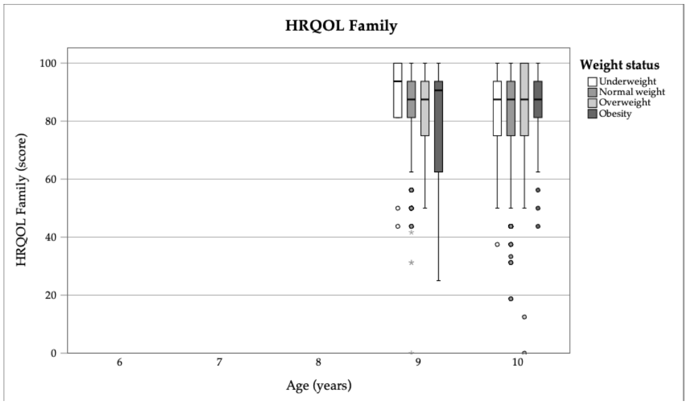


(o)

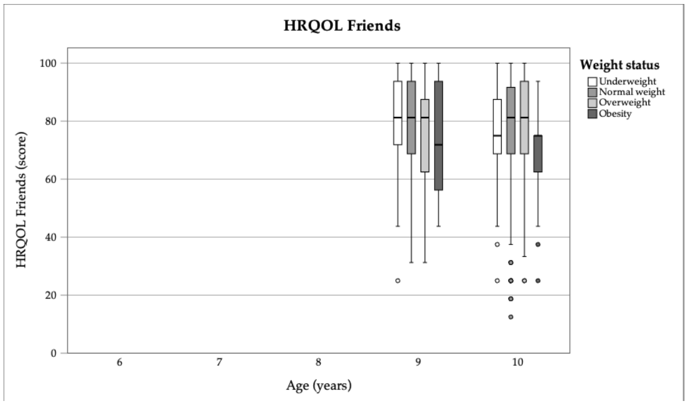


(p)

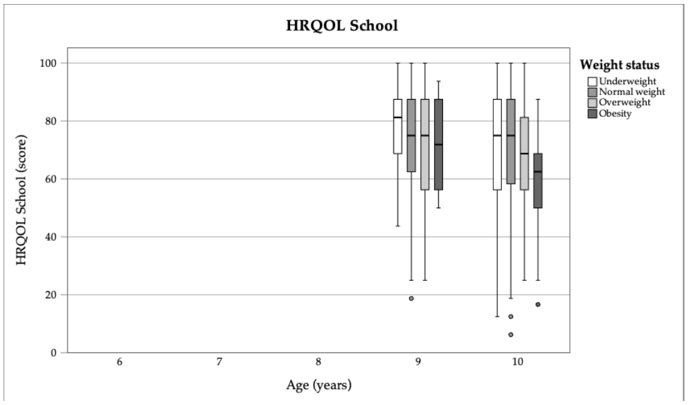


(q)

**Supplementary Figure S2.** Differences between weight classes in all parameters, (a)-(c) anthropometrics, (d) – (i) physical fitness tests, (j) concentration score, (k) – (q) health-related Quality of Life (HRQOL) sub-scales
