# Supplementary material for: A Better Cardiopulmonary Fitness Is Associated with Improved Concentration Level and Health-Related Quality of Life in Primary School Children
Source: J Clin Med. 2022 Feb 28;11(5):1326. doi: 10.3390/jcm11051326 (PMC8911456; doi:10.3390/jcm11051326)
Supplement: Supplementary file 1 [file jcm-11-01326-s001.zip › jcm-1510622-supplementary/Supplementary_new/Supplementary_Table_S1.pdf]

Supplementary Table S1. Descriptive statistics and differences between age groups total and differentiated by boys and girls in anthropometric characteristics, physical fitness parameters, concentration and health-related quality of life.

		Age (years)										
		6		7		8		9		10		
		n	M [CI 95%]	n	M [CI 95%]	n	M [CI 95%]	n	M [CI 95%]	n	M [CI 95%]	*p*
**Physical Fitness**												
Height (cm)	Total	552	123.0 [122.5, 123.4] [7,8,9,10]	1491	127.2 [126.9, 127.5] [6,8,9,10]	1596	132.9 [132.6, 133.2] [6,7,9,10]	1731	138.4 [138.1, 138.7] [6,7,8,10]	1143	143.1 [142.7, 143.5] [6,7,8,9]	<. 001*
	Girls	291	122.5 [121.9, 123.1] [7,8,9,10]	761	126.5 [126.1, 126.9] [6,8,9,10]	830	132.4 [132.0, 132.8] [6,7,9,10]	857	137.9 [137.5, 138.4] [6,7,8,10]	538	142.7 [142.1, 143.2] [6,7,8,9]	<. 001*
	Boys	261	123.6 [122.9, 124.2] [7,8,9,10]	730	127.8 [127.4, 128.3] [6,8,9,10]	766	133.4 [133.0, 133.8] [6,7,9,10]	874	138.9 [138.5, 139.3] [6,7,8,10]	605	143.6 [143.0, 144.1] [6,7,8,9]	<. 001*
Weight (kg)	Total	552	24.23 [23.86, 24.60] [7,8,9,10]	1491	26.29 [26.04, 26.54] [6,8,9,10]	1596	29.74 [29.44, 30.04] [6,7,9,10]	1731	33.25 [32.92, 33.58] [6,7,8,10]	1143	37.16 [36.65, 37.67] [6,7,8,9]	<. 001*
	Girls	291	23.87 [23.34, 24.40] [7,8,9,10]	761	25.79 [25.45, 26.14] [6,8,9,10]	830	29.45 [29.02, 29.88] [6,7,9,10]	857	32.85 [32.37, 33.32] [6,7,8,10]	538	36.44 [35.69, 37.19] [6,7,8,9]	<. 001*
	Boys	261	24.63 [24.12, 25.14] [7,8,9,10]	730	26.81 [26.44, 27.17] [6,8,9,10]	766	30.05 [29.63, 30.47] [6,7,9,10]	874	33.64 [33.19, 34.10] [6,7,8,10]	605	37.79 [37.10, 38.49] [6,7,8,9]	<. 001*
BMI (kg/m^2)	Total	552	15.93 [15.76, 16.10] [8,9,10]	1491	16.16 [16.05, 16.28] [8,9,10]	1596	16.73 [16.60, 16.85] [6,7,9,10]	1731	17.23 [17.10, 17.36] [6,7,8,10]	1143	17.99 [17.80, 18.18] [6,7,8,9]	<. 001*
	Girls	291	15.82 [15.57, 16.06] [8,9,10]	761	16.02 [15.87, 16.17] [8,9,10]	830	16.67 [16.50, 16.85] [6,7,9,10]	857	17.13 [16.95, 17.32] [6,7,8,10]	538	17.76 [17.48, 18.03] [6,7,8,9]	<. 001*
	Boys	261	16.06 [15.83, 16.29] [8,9,10]	730	16.32 [16.15, 16.48] [8,9,10]	766	16.78 [16.61, 16.96] [6,7,9,10]	874	17.33 [17.15, 17.51] [6,7,8,10]	605	18.19 [17.93, 18.45] [6,7,8,9]	<. 001*
zBMI	Total	552	0.033 [-0.046, 0.113]	1491	-0.001 [-0.052, 0.050]	1596	0.031 [-0.019, 0.081]	1731	0.030 [-0.018, 0.078]	1143	0.106 [0.043, 0.169]	.117
	Girls	291	-0.024 [-0.133, 0.085]	761	-0.068 [-0.137, 0.002]	830	-0.003 [-0.073, 0.066]	857	-0.021 [-0.091, 0.050]	538	0.008 [-0.085, 0.100]	.690
	Boys	261	0.097 [-0.020, 0.213]	730	0.069 [-0.005, 0.143]	766	0.068 [-0.004, 0.140]	874	0.080 [0.014, 0.145]	605	0.194 [0.107, 0.280]	.145
VO2max ($ml/kg/min^{-1}$)	Total	469	44.57 [44.28, 44.86] [8,9,10]	1312	45.00 [44.77, 45.23] [8,9]	1409	45.77 [45.49, 46.04] [6,7]	1549	46.08 [45.80, 46.37] [6,7,10]	1026	45.47 [45.09, 45.86] [6,9]	<. 001*
	Girls	248	44.15 [43.81, 44.48]	670	44.30 [44.05, 44.55]	747	44.77 [44.45, 45.08]	782	44.82 [44.50, 45.15]	476	44.37 [43.92, 44.82]	.129
	Boys	221	45.05 [44.57, 45.52] [8,9,10]	642	45.72 [45.34, 46.10] [8,9]	662	46.90 [46.45, 47.35] [6,7]	767	47.37 [46.91, 47.82] [6,7,10]	550	46.43 [45.83, 47.02] [6,9]	<. 001*
Pacer (laps)	Total	469	18.57 [17.76, 19.38] [7,8,9,10]	1312	22.10 [21.45, 22.75] [6,8,9,10]	1409	27.46 [26.68, 28.24] [6,7,9,10]	1549	31.47 [30.66, 32.28] [6,7,8]	1026	32.57 [31.48, 33.66] [6,7,8]	<. 001*
	Girls	248	17.35 [16.39, 18.30] [8,9,10]	670	20.15 [19.43, 20.87] [8,9,10]	747	24.61 [23.71, 25.50] [6,7,9,10]	782	27.88 [26.96, 28.81] [6,7,8]	476	29.43 [28.15, 30.70] [6,7,8]	<. 001*
	Boys	221	19.95 [18.62, 21.28] [7,8,9,10]	642	24.14 [23.05, 25.22] [6,8,9,10]	662	30.68 [29.40, 31.96] [6,7,9,10]	767	35.12 [33.83, 36.41] [6,7,8]	550	35.29 [33.61, 36.97] [6,7,8]	<. 001*
HGrel	Total	446	.416 [.405, .428] [7,8,9,10]	1215	.452 [.446, .459] [6,8,9,10]	1342	.481 [.475, .487] [6,7,9,10]	1421	.499 [.493, .504] [6,7,8]	934	.500 [.493, .507] [6,7,8]	<. 001*
	Girls	231	.390 [.374, .406] [7,8,9,10]	617	.434 [.425, .443] [6,8,9,10]	696	.458 [.450, .467] [6,7,9,10]	698	.477 [.469, .485] [6,7,8]	444	.482 [.472, .492] [6,7,8]	<. 001*
	Boys	215	.445 [.429, .461] [7,8,9,10]	598	.471 [.461, .480] [6,8,9,10]	646	.506 [.497, .515] [6,7]	723	.519 [.511, .528] [6,7]	490	.516 [.506, .526] [6,7]	<. 001*
PU (number)	Total	549	6.82 [6.17, 7.47] [7,8,9,10]	1481	8.85 [8.39, 9.31] [6,8,9,10]	1570	10.95 [10.46, 11.43] [6,7,9]	1710	12.01 [11.51, 12.52] [6,7,8]	111	11.97 [11.36, 12.59] [6,7]	<. 001*
	Girls	291	5.53 [4.76, 6.30] [7,8,9,10]	751	7.57 [7.00, 8.14] [6,9,10]	813	8.59 [8.03, 9.15] [6,10]	845	9.75 [9.16, 10.35] [6,7]	523	10.14 [9.35, 10.92] [6,7,8]	<. 001*
	Boys	258	8.28 [7.23, 9.33] [8,9,10]	730	10.17 [9.46, 10.88] [8,,9,10]	757	13.47 [12.71, 14.24] [6,7]	865	14.23 [13.44, 15.01] [6,7]	588	13.60 [12.69, 14.52] [6,7]	<. 001*

CU (number)	Total	550	10.92 [9.88, 11.97] [7,8,9,10]	1489	16.69 [15.86, 17.53] [6,8,9,10]	1582	27.89 [26.64, 29.14] [6,7,9,10]	1725	36.01 [34.63, 37.39] [6,7,8,10]	1132	39.55 [37.77, 41.34] [6,7,8,9]	<. 001*
	Girls	290	11.00 [9.72, 12.28] [7,8,9,10]	757	18.07 [16.85, 19.29] [6,8,9,10]	822	27.94 [26.22, 29.65] [6,7,9,10]	851	36.95 [34.94, 38.95] [6,7,8]	535	39.83 [37.29, 42.38] [6,7,8]	<. 001*
	Boys	260	10.84 [9.14, 12.53] [8,9,10]	732	15.27 [14.15, 16.39] [8,9,10]	760	27.85 [26.01, 29.68] [6,7,9,10]	874	35.10 [33.20, 36.99] [6,7,8,10]	597	39.30 [36.80, 41.81] [6,7,8,9]	<. 001*
SLJ (cm)	Total	550	107.2 [105.7, 108.7] [7,8,9,10]	1491	115.1 [114.1, 116.0] [6,8,9,10]	1585	124.1 [123.1, 125.1] [6,7,9,10]	1728	132.8 [131.8, 133.8] [6,7,8,10]	1136	137.1 [135.8, 138.4] [6,7,8,9]	<. 001*
	Girls	290	103.8 [102.0, 105.7] [7,8,9,10]	758	111.6 [110.3, 112.9] [6,8,9,10]	824	120.1 [118.8, 121.4] [6,7,9,10]	857	129.2 [127.9, 130.6] [6,7,8,10]	532	133.9 [132.1, 135.6] [6,7,8,9]	<. 001*
	Boys	260	111.0 [108.6, 113.3] [7,8,9,10]	733	118.6 [117.3, 120.0] [6,8,9,10]	761	128.5 [127.0, 130.0] [6,7,9,10]	871	136.3 [134.9, 137.8] [6,7,8,10]	604	140.0 [138.1, 141.8] [6,7,8,9]	<. 001*
**Concentration**												
d2-R (score)	Total			123	72.50 [69.54, 75.45] [8,9,10]	414	79.67 [77.52, 81.81] [7,9,10]	508	94.19 [92.38, 96.00] [7,8]	342	98.23 [95.56, 100.89] [7,8]	<. 001*
	Girls			67	73.90 [69.60, 78.19] [9,10]	218	80.13 [77.02, 83.24] [9,10]	257	96.99 [94.47, 99.51] [7,8]	162	100.75 [96.76, 104.73] [7,8]	<. 001*
	Boys			56	70.82 [66.74, 74.90] [9,10]	196	79.15 [76.19, 82.11] [9,10]	251	91.32 [88.75, 93.90] [7,8]	180	95.96 [92.37, 99.54] [7,8]	<. 001*
**HRQOL**												
HRQOL Total (score)	Total							326	76.73 [75.63, 77.83]	672	75.78 [74.98, 76.58]	.179
	Girls							176	77.06 [75.68, 78.44]	339	77.08 [75.97, 78.19]	.987
	Boys							150	76.34 [74.55, 78.12]	333	74.46 [73.31, 75.61]	.066
Physical well-being (score)	Total							326	79.52 [77.87, 81.18]	672	79.00 [77.82, 80.17]	.612
	Girls							176	79.73 [77.59, 81.88]	339	78.60 [76.92, 80.28]	.429
	Boys							150	79.28 [76.70, 81.86]	333	79.40 [77.74, 81.06]	.937
Emotional well-being (score)	Total							326	83.94 [82.66, 85.22] [10]	672	82.17 [81.11, 83.22] [9]	.036*
	Girls							176	83.53 [81.89, 85.18]	339	83.14 [81.71, 84.58]	.751
	Boys							150	84.42 [82.40, 86.44] [10]	333	81.17 [79.62, 82.73] [9]	.013*
Self-esteem (score)	Total							326	61.73 [59.48, 63.99]	672	61.61 [60.07, 63.15]	.929
	Girls							176	61.49 [58.74, 64.25]	339	62.86 [60.75, 64.97]	.474
	Boys							150	62.01 [58.28, 65.75]	333	60.34 [58.09, 62.60]	.406
Family (score)	Total							326	84.33 [82.63, 86.03]	672	84.09 [82.88, 85.29]	.819
	Girls							176	86.62 [84.83, 88.41]	339	86.20 [84.68, 87.71]	.769
	Boys							150	81.64 [78.64, 84.64]	333	81.94 [80.09, 83.79]	.846
Friends (score)	Total							326	76.83 [75.02, 78.65]	672	77.24 [75.95, 78.52]	.722
	Girls							176	76.66 [74.22, 79.09]	339	78.88 [77.11, 80.64]	.155
	Boys							150	77.04 [74.30, 79.78]	333	75.57 [73.71, 77.43]	.373

School (score)	Total		326	73.84 [71.99, 75.70] [10]	672	70.50 [69.09, 71.90] [9]	.005*
	Girls		176	74.11 [71.61, 76.61]	339	72.62 [70.65, 74.58]	.372
	Boys		150	73.53 [70.73, 76.33] [10]	333	68.34 [66.33, 70.34] [9]	.003*

Values are number or mean [CI 95%]. BMI = body mass index, zBMI = standardized BMI, HGrel = handgrip strength relativized to body weight, PU = Push-Ups, CU = Curl-Ups, SLJ = Standing long jump, d2-R = concentration test, HRQOL = health-related quality of life; * $p < .05$, [6 7 8 9 10] = significant post-hoc differences between age groups
