# Supplementary material for: A Better Cardiopulmonary Fitness Is Associated with Improved Concentration Level and Health-Related Quality of Life in Primary School Children
Source: J Clin Med. 2022 Feb 28;11(5):1326. doi: 10.3390/jcm11051326 (PMC8911456; doi:10.3390/jcm11051326)
Supplement: Supplementary file 1 [file jcm-11-01326-s001.zip › jcm-1510622-supplementary/Supplementary_new/Supplementary_Table_S2.pdf]

Supplementary Table S2. Multiple linear regression analyses between HRQOL sub-scales and physical fitness parameters.

Coefficients	*b* [95% CI$_b$][a]	Se$_b$[a]	*β*	*t*	*p*
Dependent Variable: **Physical well-being**					
BMI	.119 [-.309, .569]	0.23	0.02	0.571	.568
VO2max	.384 [.167, .589]	0.11	0.16	3.537	< .001 *
HGrel	.377 [-12.864, 12.953]	6.379	0.00	0.063	.950
PU	.115 [-.021, .253]	0.07	0.08	1.803	.072
CU	.047 [.013, .081]	0.02	0.10	2.664	.008 *
SLJ	.036 [-.034, .107]	0.04	0.05	1.045	.297
Dependent Variable: **Emotional well-being**					
Age	-2.979 [-5.587, -.237]	1.340	-0.08	-2.345	.019 *
BMI	-.025 [-.383, .350]	0.18	-0.01	-0.141	.888
VO2max	.143 [-.041, .324]	0.10	0.07	1.554	.121
HGrel	-2.363 [-12.621, 8.156]	5.162	-0.02	-0.469	.639
PU	.046 [-.067, .153]	0.05	0.04	0.846	.398
CU	.020 [-.010, .050]	0.01	0.05	1.351	.177
SLJ	.056 [-.001, .113]	0.03	0.09	1.926	.054
Dependent Variable: **Self-esteem**					
BMI	.043 [-.499, .579]	0.27	0.01	0.155	.877
VO2max	.349 [.064, .635]	0.14	0.11	2.389	.017 *
HGrel	29.687 [13.509, 44.924]	7.743	0.16	3.698	< .001 *
PU	-.038 [-.193, .115]	0.08	-0.02	-0.437	.662
CU	.035 [-.010, .079]	0.02	0.05	1.473	.141
SLJ	-.058 [-.143, .029]	0.05	-0.06	-1.261	.208
Dependent Variable: **Family**					
Sex	4.214 [2.035, 6.430]	1.091	0.14	3.938	< .001 *
CU	.032 [-.001, .066]	0.02	0.07	1.919	.055
Dependent Variable: **Friends**					
BMI	-.017 [-.488, .499]	0.23	-0.00	-0.075	.940
VO2max	.339 [.125, .565]	0.12	0.13	2.829	.005 *
HGrel	-2.858 [-15.908, 9.980]	6.723	-0.02	-0.433	.665
PU	.014 [-.129, .163]	0.07	0.01	0.200	.842
CU	.059 [.021, .097]	0.02	0.11	2.991	.003 *
SLJ	.018 [-.047, .085]	0.03	0.02	0.477	.634
Dependent Variable: **School**					
Age	-2.681 [-6.100, .844]	1.703	-0.05	-1.518	.129
BMI	-.664 [-1.177, -.152]	0.26	-0.11	-2.694	.007 *
VO2max	.518 [.249, .775]	0.13	0.19	4.056	< .001 *
HGrel	-6.923 [-21.203, 6.986]	6.985	-0.04	-0.989	.323
PU	.063 [-.087, .215]	0.08	0.04	0.847	.397
CU	.041 [.004, .081]	0.02	0.07	1.983	.048 *
SLJ	-.042 [-.124, .039]	0.04	-0.05	-1.053	.293

[a] Se$_b$ = Confidence interval and Coefficients Standard Error per BCa-Bootstrapping with 2.000 BCa samples; ß = standardized ß, BMI = body mass index, HGrel = handgrip strength relativized to body mass, PU = Push-Ups, CU = Curl-Ups, SLJ = Standing long jump, HRQOL total = KINDL total score; * $p < .05$
